# Performance against quality indicators in the initial assessment of patients with respiratory infections in acute medicine services

**DOI:** 10.1136/bmjresp-2025-003207

**Published:** 2025-08-26

**Authors:** Catherine Atkin, Mark Holland, Tim Cooksley, Ragit Varia, Christian P Subbe, Tom Wilkinson, Daniel Lasserson, Elizabeth Sapey

**Affiliations:** 1 Inflammation and Ageing, School of Infection, Inflammation and Immunology, University of Birmingham, Birmingham, UK; 2 Acute Medicine, University Hospitals Birmingham NHS Foundation Trust, Birmingham, UK; 3 Department of Clinical and Biomedical Sciences, University of Greater Manchester, Bolton, UK; 4 Acute Medicine, Manchester University NHS Foundation Trust, Manchester, UK; 5 The Christie NHS Foundation Trust, Manchester, UK; 6 Acute Medicine, Mersey and West Lancashire Teaching Hospitals NHS Trust, Prescot, UK; 7 Ysbyty Gwynedd, Bangor, UK; 8 School of Medical Sciences, Bangor University, Bangor, UK; 9 Clinical and Experimental Sciences, Faculty of Medicine, University of Southampton, Southampton, UK; 10 Warwick Medical School, University of Warwick, Coventry, UK; 11 Department of Geriatric Medicine, John Radcliffe Hospital, Oxford, UK; 12 NIHR Midlands Patient Safety Research Collaboration, University of Birmingham, Birmingham, UK; 13 NIHR Birmingham Biomedical Research Centre, University of Birmingham, Birmingham, UK; 14 School of Medical Sciences, University of Birmingham, Birmingham, UK

**Keywords:** Pneumonia, Respiratory Infection

## Abstract

**Introduction:**

Hospital attendances due to respiratory infection peak in winter, contributing to pressures within acute services. We assessed the prevalence of suspected respiratory infection within acute medical admissions during winter and evaluated performance against recommendations for initial assessment.

**Methods:**

Data were collected through the Society for Acute Medicine (SAM) Benchmarking Audit, comprising a hospital-level survey and 24-hour patient-level data collection for unplanned acute medical attendances on 22 February 2024. Performance metrics assessed included those from the SAM’s clinical quality indicators (CQI) for medical admissions, and British Thoracic Society (BTS) guidelines for community acquired pneumonia.

**Results:**

Data were available for 4390 patients at 76 hospitals. Suspected respiratory infections accounted for 22.8% of all unplanned medical attendances; these patients were older (age ≥70 years: 58.2% vs 44.7%, p<0.001) and had higher National Early Warning Score 2 (NEWS2) scores (NEWS2 ≥3: 63.8% vs 23.8%, p<0.001) than those without respiratory infection; they were more likely to be assessed in the emergency department (80.8% vs 63.7%, p<0.001), and had lower rates of discharge without overnight admission (14.9% vs 35.9%, p<0.001). 71.0% of patients underwent a chest X-ray within 4 hours of arrival; 27.0% were reported within 12 hours. Antibiotics were administered ≥4 hours from arrival in 32.9%. Performance against these indicators varied between hospitals. Nine hospitals (12.7%) had a separate respiratory admission service; this was not associated with improved performance against SAM CQIs or BTS guidance.

**Conclusion:**

Respiratory infections contribute significantly to acute medical attendances via the emergency department. There remains significant scope to improve key steps in initial assessment and management.

**WHAT IS ALREADY KNOWN ON THIS TOPIC**

- Respiratory infections are a leading cause of hospital attendance and admission, with seasonal variation that peaks in winter. A British Thoracic Society audit in 2019 demonstrated issues with delivery of targets for initial assessment of patients with community-acquired pneumonia, but included only those with a primary diagnosis of pneumonia on clinical coding.

**WHAT THIS STUDY ADDS**

- This study demonstrates the proportion of medical attendances to hospital that are suspected to have a respiratory infection and demonstrates that these patients are more likely to be high acuity and require prolonged inpatient admission. Performance against assessment pathway targets, such as imaging and antibiotics, shows ongoing deficits in performance, varying between hospitals.

**HOW THIS STUDY MIGHT AFFECT RESEARCH, PRACTICE OR POLICY**

- Further research should explore reasons for variation in performance, and how high performance can be achieved consistently across National Health Service hospitals.

## Introduction

 Respiratory infections are an important cause of attendances to urgent and emergency care services, and of emergency hospital admissions, causing more than 570 000 emergency admissions in England annually.^1^ Peaks of respiratory infection, including respiratory viruses, occur during winter months,^2^ and hospital admissions due to respiratory disease are 80% higher in January than in summer.^3^ With the rising pressures now seen within urgent and emergency care services across the UK, it is increasingly important to understand how these common conditions contribute to the acute medical admissions.

Community-acquired pneumonia (CAP) remains an important cause of morbidity and mortality in the UK, emphasised within a recent National Confidential Enquiry into Patient Outcome and Death (NCEPOD) report, released in December 2023.^4^ This report, alongside national guidance,^5^,^7^ highlights the importance of appropriate assessment and management within the first 4 hours following hospital arrival. In the UK, this is delivered by emergency medicine for those attending via the emergency department (ED), and by acute and general medicine teams, who provide specialist assessment and management of internal medicine patients referred from emergency medicine, primary care and other community sources.^8^ This includes management of those requiring hospital admission through acute medical units (AMUs), as well as assessment and management without overnight admission through same day emergency care (SDEC) services.^9^

Although admissions for CAP have previously been audited nationally via the British Thoracic Society (BTS),^10^ this audit assessed only diagnoses that were recorded through clinical coding and the overall contribution of respiratory infection to acute medical attendances, and performance against quality indicators for their initial assessment has not been reported since the start of the COVID-19 pandemic. We aimed to assess the impact of respiratory infections on unplanned medical attendances in the UK during winter and to evaluate delivery of recommended standards for initial assessment and management for suspected respiratory infection, using the Society for Acute Medicine (SAM) Benchmarking Audit (SAMBA).

## Methods

Winter SAMBA24 (wSAMBA24) took place on 22 February 2024. Participation was open to all hospitals accepting unplanned admissions to internal medicine (including acute and/or general medicine); community hospitals were excluded. Participating sites registered the audit locally, obtaining appropriate approvals; the wSAMBA24 protocol is available online.^11^ Data was uploaded to a REDCap (Research Electronic Data Capture) database, hosted at the University of Birmingham.^12^ Health Research Authority approval has been granted to allow secondary analysis on non-identifiable data (REC 21/HRA/4196).

Hospital-level data comprised a survey describing acute medicine service structure, including hospital size assessed by total number of inpatient beds. Patient-level data was collected for all medical attendances arriving to hospital within a 24-hour period (00:00–23:59), describing demographic variables and processes of care. Data collectors were asked to collect data as early as possible (preferably within 12 hours of admission), using routinely collected data from clinical records and patient administration systems (PASs), with follow-up and discharge data collected from PAS or clinical records after 7 days, and data uploaded within the following 6 weeks.

The primary aim of SAMBA is to assess performance against the SAM’s clinical quality indicators (CQIs) for acute medicine, based on recommendations from the National Institute for Health and Care Excellence (NICE). These are outlined in table 1.

**Table 1:** Standards assessed within Winter SAMBA24

Acute medicine clinical quality indicators	Early warning score recorded within 30 minutes of arrival. Assessment by a competent clinical decision maker within 4 hours of arrival. Review by consultant physician within target time: 6 hours for patients arriving between 08:00 and 19:59. 14 hours for patients arriving between 20:00 and 07:59.
Standards from community acquired pneumonia guidance	A CURB65 score should be calculated.* Chest X-ray should be performed within 4 hours of hospital arrival.† Formal chest X-ray report available within 12 hours of the X-ray.† Antibiotic therapy started within 4 hours of arrival.* Oxygen should be prescribed with an appropriate target range.

* For patients with community acquired pneumonia (CAP).

† For patients with suspected CAP.

CURB65, Confusion, blood Urea, Respiratory rate, Blood pressure, age ≥65 years ; SAMBA, Society for Acute Medicine Benchmarking Audit.

### Patient and public involvement

There was no specific public or patient involvement in the design of SAMBA.

Questions were included in both the hospital-level and patient-level survey regarding respiratory infections (online supplemental box 1). These questions were based on recommendations for CAP from the December 2023 NCEPOD report^1 2^ as well as guidelines from the BTS and NICE.^6 7 13^ The chosen standards from these guidelines are included in table 1. Patients with suspected respiratory infection were identified by the data collectors at each site; this was further subdivided by ‘type’ of respiratory infection. More than one infection type could be recorded; for comparison between infection types, CAP was assumed to be the primary diagnosis where selected.

The first early warning score on arrival to hospital was documented using National Early Warning Score 2 (NEWS2).^14^ For patients with CAP, the CURB65 score (confusion, blood urea, respiratory rate, blood pressure, age ≥65 years) was recorded.^15^

Hospitals were excluded from the analysis described here if they were: non-standard acute medicine units (eg, frailty specific services), located outside the northern hemisphere and therefore not in winter, or where the question identifying whether the patient had a suspected respiratory infection, and therefore all responses related to respiratory infection, was missing data for more than 40% of patients, due to inability to assess prevalence of respiratory infection.

To compare hospital-level data factors by size of hospitals, respondents were divided into three equal groups: small (<390 inpatient beds), medium (390–625 inpatient beds) and large (>625 inpatient beds).

Descriptive statistics were calculated using Stata/SE V.18.0. Data are summarised with mean and SD where normally distributed, and otherwise as median and IQR. Comparison of performance between dichotomous variables was performed using χ², or Fisher’s exact test where expected cell counts were less than 5. For continuous variables, independent samples t-test was used for variables that were normally distributed, and Kruskal-Wallis (KW) test was used for those that were not; normality was assessed using Shapiro-Wilk normality test. Multivariable analysis was performed using logistic regression; variables included within models were selected based on previous analyses of similar data sets.^16^ A p value of<0.05 was used to signify statistical significance throughout.

## Results

81 hospitals participated in wSAMBA24. Five hospitals were excluded from this analysis (online supplemental figure 1). Patient level data regarding respiratory infections was therefore available for 4390 unplanned adult medical attendances, at 76 acute medicine services. Of these hospitals, 71 had submitted hospital-level data related to respiratory infection.

Hospital size ranged from 175 to 1300 beds (median 508, IQR 382–705). 93.4% of participating units (71 hospitals) were in England, 2.6% (2 hospitals) were in British Crown Dependencies, 2.6% (2 hospitals) were in Northern Ireland and 1.3% (1 hospital) was in Scotland.

### Hospital-level data

Of the 71 hospitals submitting both hospital and patient level data, 12.7% (9 hospitals) had a separate respiratory admission service; this operated 24/7 in 4.2% (3 hospitals) and for restricted hours in 8.5% (6 hospitals). There were no small or medium hospitals that offered a 24/7 separate respiratory admission service. Larger hospitals were more likely to have a separate respiratory admission service than medium or small hospitals (Fisher’s exact p=0.015).

A local guideline regarding CAP was available in 94.4% of hospitals; this was a guideline specifically for CAP in 57.8% and part of a wider antibiotic guideline in the remainder. A CAP care bundle was used by 33.8% of hospitals. There was no difference in guideline or care bundle availability compared by hospital size (table 2). Written information for patients with CAP was provided on discharge from hospital in 11.3%. Availability of point of care testing for respiratory viruses is also shown in table 2. 53 hospitals (74.7%) had point of care testing for COVID-19, 47 (66.1%) for influenza viruses and 26 (36.6%) for respiratory syncytial virus.

**Table 2:** Hospital level service availability, for services related to respiratory infection, by hospital size

	Overall	Small	Medium	Large	P value
	% (N)	% (N)	% (N)	% (N)	
Respiratory admission pathway	12.7 (9/71)	4.2 (1/23)	8.3 (1/24)	29.2 (7/24)	0.015*
CAP guideline available	94.4 (67/71)	87.0 (20/23)	95.8 (23/24)	100 (24/24)	0.118*
Specific guideline	57.8 (41/71)	47.8 (11/23)	66.7 (16/24)	58.3 (14/24)	
As part of wider guideline	36.6 (26/71)	39.1 (9/23)	29.2 (7/24)	41.7 (10/24)	
CAP care bundle in use	33.8 (24/71)	34.8 (8/23)	29.2 (7/24)	37.5 (9/24)	0.711
Written information regarding CAP provided on discharge	11.3 (8/71)	13.0 (3/23)	16.7 (4/24)	4.2 (1/24)	0.421*
Point of care respiratory virus testing	74.7 (53/71)	78.3 (18/23)	58.3 (14/24)	87.5 (21/24)	0.076*
Influenza	66.2 (47/71)	73.9 (17/23)	50.0 (12/24)	75.0 (18/24)	0.119
COVID-19	74.7 (53/71)	78.3 (18/23)	58.3 (14/24)	87.5 (21/24)	0.076
Respiratory syncytial virus (RSV)	36.6 (26/71)	39.1 (9/23)	29.2 (7/24)	41.7 (10/24)	0.677

* P value for Fisher's exact test; χ² used for all others.

CAP, community acquired pneumonia.

### Patient-level data

Of the 4390 unplanned admissions, the presentation was due to a suspected respiratory infection in 22.8% (988 patients). The proportion of patients with suspected respiratory infection varied between hospitals (median 25.0%, IQR 17.6–27.9%, range 6.5–41.9%), and within each assessment location (ED: 27.4%, AMU: 25.0%, SDEC: 11.7%, p<0.001). CAP was the most common type of respiratory infection (372 patients, 37.7%), followed by ‘other lower respiratory tract infection (LRTI)’ (326, 33.0%), infective exacerbation of chronic respiratory disease (223, 22.6%), respiratory virus infection with confirmatory result (66, 6.7%) and hospital-acquired pneumonia (HAP) (27, 2.7%). 26 patients (2.6%) had more than one diagnosis selected.

Demographics of those with suspected respiratory infection were compared with those without (table 3). Patients with respiratory infections were older; 28.0% of those aged over 70 years had a respiratory infection, compared with 18.4% of those aged under 70 years (p<0.005). There was a higher rate of respiratory infection in women compared with men (24.7% vs 21.2%, p=0.006), and in care home residents (39.4% vs 22.1%, p<0.001). This difference persisted after adjusting for age (adjusted OR (AOR) 1.88, 95% CI 1.41 to 2.51, p<0.001). Comparisons between respiratory infection diagnoses are shown in online supplemental table 1. Patients with HAP were more likely to be aged over 70 and resident in a care home.

**Table 3:** Comparison of patient characteristics and demographics of unplanned medical attendances with versus without suspected respiratory infection

	Suspected respiratory infection (n=988)		No suspected respiratory infection (n=3306)		P value
	%	N	%	N	
Age					<0.001*
16–19	0.8	8	1.8	58	
20–29	2.9	29	7.1	235	
30–39	4.9	48	9.3	306	
40–49	5.6	55	8.9	295	
50–59	10.2	101	12.9	427	
60–69	17.3	171	15.4	508	
70–79	26.7	264	19.8	654	
80–89	23.2	229	19.4	640	
90+	8.3	81	5.5	183	
Missing		1			
Aged≥70 years	58.2	575	44.7	1477	<0.001
Gender					0.006
Male (%)	43.1	426	48.2	1594	
Missing		1			
Care home residence	9.3	92	4.3	141	<0.001
Missing		2		6	
Hospital discharge within previous 30 days	22.6	223	19.6	645	0.04
Missing		3		22	
NEWS2 on arrival					<0.001*
0	10.8	97	37.3	1122	
1	11.4	103	24.5	739	
2	14.0	126	14.4	435	
3	12.8	118	9.1	274	
4	13.1	115	5.4	164	
5	12.8	87	3.5	104	
6	9.7	70	2.3	70	
7+	20.5	185	3.5	104	
Missing		87		294	
≥3	63.8	575	23.8	716	<0.001
Daytime arrival (08:00–19:59)	74.9	740	77.5	2562	0.089
Source of referral					<0.001
ED	66.0	652	60.8	2009	
GP	17.3	171	21.4	707	
Paramedic	11.3	112	8.2	271	
Other	5.4	53	9.7	319	
Location of first clinical assessment					<0.001
ED	80.8	792	63.7	2096	
AMU	5.8	57	5.2	171	
SDEC	13.2	130	29.8	983	
Other	0.1	1	1.2	39	
Missing		8		17	

* P values for Kruskal-Wallis test; χ² used for all others

AMU, acute medical unit; ED, emergency department; GP, general practice; NEWS2, National Early Warning Score 2; SDEC, same day emergency care.

### Pathways through acute services

Patients with respiratory infections were more likely to receive their initial assessment in the ED (p<0.001). They were also more likely to have an elevated NEWS2 score on arrival to hospital (KW p<0.001, table 3). Within each assessment location, patients with respiratory infection were more likely to have a raised NEWS2 than those without respiratory infection (NEWS2 ≥3 by location of initial assessment: ED: 70.9% vs 31.5%, p<0.001; AMU: 50.9% vs 22.1%, p<0.001; SDEC: 40.6% vs 7.4%; p<0.001).

Initial clinician assessment occurred within 4 hours in 80.1% of patients with respiratory infection, and consultant review occurred within target times in 49.5%. Using SAM’s CQIs as a marker for speed of assessment through the admissions pathway, suspected respiratory infection did not appear to impact likelihood of achieving assessment within target time frames (online supplemental table 2).

### Standards for assessment of patients with respiratory infection

Performance against the selected targets related to respiratory infection is shown in table 4. A chest X-ray was performed for 95% of patients with respiratory infection; 51 patients did not have a chest X-ray.

**Table 4:** Performance against standards for assessment and management of acute respiratory infection (community-acquired pneumonia (CAP))

	Performance (overall)		Performance by hospital	
	%	N	Median	IQR (range)
CURB65 documented*	37.9	139/367	37.5%	0–50.0% (0–100%)
Chest X-ray within 4 hours†	71.0	651/917	75.0%	63.8–84.9% (22.2–100%)
Chest X-ray report within 12 hours†	27.0	249/921	16.7%	0–45.2% (0–100%)
Antibiotics within 4 hours	67.1	496/739	67.9%	55.4–83.3% (0–100%)
Administered oxygen was prescribed	66.3	317/478	77.8%	50.0–100% (0–100%)

Performance for all included patients, and hospital-level performance by participating hospital site.

* For patients with CAP diagnosis only.

† Target applied to patients who had chest X-ray performed.

CURB65, confusion, blood urea, respiratory rate, blood pressure, age ≥65 years.

More than half (50.4%, 493/979) of patients with suspected respiratory infection received supplementary oxygen prior to consultant physician review (CAP: 57.3%, other LRTI: 38.2%, infective exacerbations: 56.9%, respiratory viruses: 45.2%, HAP: 61.5%, p<0.001). Antibiotics were prescribed for 89.1% of patients with suspected respiratory infection (875/982). Of those receiving antibiotics, the first dose was given within 2 hours of hospital arrival in 39.5%, 2–4 hours in 27.6%, 4–8 hours in 20.3% and more than 8 hours from arrival in 12.6%.

CURB65 scores were documented for 139 patients with CAP; however, four values were missing from analysis. Of those with a documented score, 43.7% (59 patients) had a score of 0–1, 23.0% (31 patients) scored 2 and 33.3% (45 patients) had a score ≥3. 95.6% of those with a CURB65 score ≥3 received their first dose of antibiotics intravenously, compared with 80.7% of those with a score of 2 and 71.2% of those with a score of 0–1 (p=0.007).

A higher NEWS2 value on arrival to hospital was associated with a greater likelihood of achieving antibiotic delivery and of chest X-ray acquisition within 4 hours (online supplemental table 3). This was also influenced by time of hospital arrival, and the location of the first clinical assessment, with targets less likely to be met for patients receiving their first assessment in AMU. Oxygen prescription in those receiving oxygen did not appear to be associated with these factors.

Performance against respiratory infection targets for patients with CAP compared with patients with diagnoses other than CAP is shown in online supplemental table 4. There was no difference in performance comparing patients with CAP to other diagnoses. Of those with CAP receiving supplemental oxygen (186 patients), 19.9% (37 patients) achieved all the CAP care bundle targets.

There were 4 hospitals (5.3%) where all patients with CAP had a CURB65 score documented, 9 hospitals (11.8%) where all chest X-rays were performed within 4 hours of arrival, 10 hospitals (13.2%) where all the patients who were prescribed antibiotics received this within 4 hours of arrival and 32 hospitals (42.7%) where all patients who received supplemental oxygen had this prescribed. There were no hospitals that achieved 100% across all targets.

Performance against these targets was assessed for hospitals with a separate respiratory admissions pathway compared with those without. There was no difference in performance comparing those hospitals with and without a respiratory admission pathway.

### Outcomes

Of those with respiratory infection, 14.9% were discharged without overnight admission; this was lower than those without (35.9%, p<0.001). The rate of same-day discharge was higher in patients with LRTI than with CAP (28.3% vs 9.1%, p<0.001), with 15.3% of those with CURB65 0–1 discharged the same day; no patients with HAP were discharged the same day. Same day discharge was less likely in those with respiratory infection diagnoses, adjusting for known influencing factors (online supplemental table 5, AOR CAP 0.35, 95% CI 0.22 to 0.54, p<0.001). In those with respiratory infection, 32.2% of discharges occurred from non-SDEC locations.

During their admission, 34.3% of patients with a respiratory infection (334/974) were seen by a respiratory specialist. Only six patients with suspected respiratory infection who were discharged without overnight admission were reviewed by a respiratory physician (4.2%). The rate of same-day discharge for patients with suspected respiratory infection was no different comparing hospitals with a respiratory admission service to those without (Kruskal-Wallis, p=0.463).

Patients with respiratory infection were more likely to be in hospital at 7 days (31.5% vs 21.7%, p<0.001), and had a higher 7-day mortality rate (4.7% vs 1.9%, p<0.001). Seven-day mortality varied between diagnoses (CAP: 5.9%; other LRTI: 3.8%; infective exacerbation of respiratory infection: 3.0%; respiratory viruses: 3.3%; HAP: 15.4%; p=0.041).

## Discussion

These results demonstrate that respiratory infection contributes a significant proportion of unplanned medical attendances to hospital, with more than a quarter of acute medical patients assessed via ED pathways attending with a respiratory infection. With this impact on the acute and general medicine workload, and targets for assessment and management of those with respiratory infection directed at the first few key hours following arrival to hospital, this underscores the importance of ensuring robust clinical management of these patients by acute medical teams.

Patients with respiratory infections mainly presented via ED attendance and had higher acuity, on average, compared with medical patients without respiratory infection. Respiratory infection in itself did not appear to influence speed of clinical assessment, as previously demonstrated with other conditions,^17^ after accounting for acuity assessed by NEWS2. As 20% of medical patients waited more than 4 hours for clinician assessment, this likely impacts performance against time to chest X-ray and antibiotic, with performance relying on identification during initial triage and rapid assessment and treatment processes.^18^

All standards related to respiratory infection were achieved for less than 75% of patients in this analysis. Performance varied between hospitals; however, there were no hospitals achieving 100% across all targets, suggesting universal scope to improve assessment pathways for patients with respiratory infection, including those with CAP, where significant morbidity and mortality is still seen.^4^ Less than 70% of patients with CAP received antibiotics within 4 hours of arrival; this remains below the aim of 85% by 2022 set following the last BTS audit of CAP management.^10^ In patients with a CURB65 score of 0–1, where oral antibiotics are consistently recommended as first line treatment, more than 70% received their first dose of antibiotics intravenously; factors driving the continued use of intravenous antibiotics in low severity patients requires further exploration if appropriate antimicrobial stewardship is to be achieved.^19^

Service level mechanisms to support delivery of CAP care pathways varied between hospitals. Although most hospitals had clinical guidelines available for CAP, this was often within wider antibiotic guidelines. The use of a CAP care bundle locally was much lower, with only a third of hospitals reporting use of a CAP care bundle, similar to the proportion reported in 2019.^10^ Care bundles are recommended for use in CAP, due to the demonstrated improvements in clinical care quality associated with care bundle implementation.^5 20 21^ A quarter of participating hospitals did not have point of care testing for respiratory viruses, despite beneficial impacts on infection control, length of stay and antimicrobial stewardship.^322^,^24^ Only 11% of hospitals provided written discharge information to patients with CAP. Our analysis focused on acute medicine services; however, even if higher rates of written information are provided for those managed on respiratory medicine wards, there remains an ongoing gap in management for patients discharged same-day or managed in non-specialty services.^10^

A separate respiratory admission service was reported by 13% of participating hospitals, similar to that reported in previous surveys (12.7–15.9%),^3 10^ and was more common in large hospitals, likely reflecting a greater number of suitable patients as well as greater resource availability. Hours of service operation varied, with one third of these services operating 24/7; however, in-depth exploration of how these services operated was beyond the scope of this study. There was no difference in performance against the indicators assessed here comparing hospitals with and without a separate respiratory admission service, suggesting further exploration of how effective implementation of these services could improve clinical care is needed, including whether there is benefit for selected cohorts of patients with respiratory conditions. The respiratory medicine Getting It Right First Time report recommends embedding respiratory teams in acute medicine to improve discharge rates, reporting analysis showing higher same-day discharge rates for patients with respiratory conditions in centres with greater respiratory consultant time provided to acute and general medicine.^3^ This could not be demonstrated within our data set, with only 1.8% of patients assessed by a respiratory physician discharged the same day. We are unable to assess whether these patients would otherwise have been admitted; however, greater respiratory medicine engagement with acute/general medicine may improve discharge rates via other methods, such as improved clinical pathways, rather than at individual patient level, or may have additional benefit in non-infectious respiratory disease.

Associations between performance against assessment time targets and assessment location may be due to differences in process, delays in movement between pathway steps or availability of staff. Caution is needed when interpreting these results, as these processes and pathways cannot be assumed to be identical between units. However, these results suggest that patients admitted directly to acute medicine units may have difficulty accessing timely diagnostic imaging, and services should ensure these patients are not disadvantaged in comparison to those presenting through emergency medicine or SDEC services; individual acute medicine services may benefit from assessing whether delays occur within different pathways within their own services and the subsequent impact on patient care, to allow targeted quality improvement interventions.

Standards for initial assessment and management of respiratory infection assessed here were chosen from relevant national guidance for CAP.^3 4 6 7^ Current definitions of CAP within the hospital setting include new radiographic changes consistent with infection.^6^ Incorporating criteria requiring review of chest X-ray findings was beyond the scope of SAMBA; similarly, reviewing whether type of respiratory infection was correctly recorded was not possible, with data collected by a range of clinicians and non-clinicians, from multiple professional backgrounds. Patient-level data in this analysis is assumed to reflect documented clinical diagnosis, which may differ from the diagnosis recorded via clinical coding, although this has previously been recognised as inaccurate in a proportion of patients,^3^ and previous audits have shown that many patients treated for CAP do not meet radiological criteria.^10^ As such, some patients identified here as CAP may not meet more stringent diagnostic criteria. Except for CURB65 score documentation, management standards were applied across respiratory infections. To reliably achieve these targets in patients with CAP would necessitate delivery in all those where CAP has not yet been excluded, as these targets include the imaging necessary to confirm or exclude the diagnosis. Performance in delivery of these standards was not significantly different in patients with CAP compared with those with other respiratory infection diagnoses, supporting this approach.

The data presented here represents a 24-hour period, and variation can be expected across time periods. This includes variation between years; rates of hospital admission for influenza in 2024 have already surpassed the corresponding 2023 data, with subsequent impacts on urgent and emergency care performance and increased pressures on ward and critical care beds.^25 26^ This increase in attendances may lead to worse performance compared with that reported here. Variation from day to day may explain in part the wide range in proportion of admissions that were identified as suspected respiratory infection; however, as secondary data quality checking was not possible, this may have been influenced by differences in data collection processes between sites.

There may be systematic differences between the hospitals that participated in this analysis and those that did not. No hospitals in Wales participated, likely due to concurrent industrial action on the day of data collection.^27^ There are approximately 225 AMUs in the UK,^16^ and this analysis therefore reflects less than half of eligible units; the participation rate was lower than previous rounds of SAMBA, likely due to additional winter pressure workload impacting staff availability. Hospitals anticipating higher clinical pressures may have been less likely to participate, in which case performance against CQIs may be an overestimate of national performance; similarly, hospitals that did not participate due to inadequate resources to deliver the audit, or those with less engagement in national audits generally, may be expected to have poorer performance than reported here.^28^ Although our findings are broadly in agreement with previous reports that respiratory illnesses account for one-third of hospital admissions,^3^ this may not reflect the peak burden of respiratory infection admissions. Dates were chosen to avoid the absolute peak of winter-related clinical pressures, to reduce impact on participation rates as much as possible, and national surveillance reported a decrease in viral respiratory infections preceding this data collection; therefore, our analysis likely underestimates peak burden.^3 29^

Service questions focused on areas that could be delivered within acute or general medicine. As such, we did not specifically ask regarding respiratory medicine services, such as respiratory infection nurse specialists, or adaptations to mitigate increased service pressures, such as expanded respiratory medicine bed base.^3^ Logistic regression was adjusted for factors that are known to influence same day discharge in the SAMBA data set, however, there may be additional contributing factors, for example, comorbidity^30^ and deprivation^3^ that are not measured in this SAMBA data set. Patients discharged directly from emergency medicine without referral to acute/general internal medicine were not included in this analysis, and overall referral rates vary between hospitals, influenced by patient demography and systems factors such as access to primary care.^31^ Although patients with respiratory infection were shown to have an average increased length of stay in comparison to those without respiratory infection, a comprehensive estimate of total length of stay could not be achieved in SAMBA as the follow-up period was limited to 7 days, shorter than median length of stay for pneumonia admissions reported previously.^3^

Given the significant number of unplanned attendances associated with respiratory infection, the ongoing associated morbidity and mortality and the variable performance in delivery of key aspects of care, an improved understanding of how care can be delivered effectively for these patients is needed. This may benefit from a national programme of audit and quality improvement, targeting the achievement of current standards and evaluating their impact on morbidity and mortality, alongside robust research designed to understand factors driving inequality in care delivery and identify optimal models of care.

## Conclusion

Respiratory infections contribute significantly to medical attendances and admissions to hospital, particularly via the ED. There remains significant room to improve key steps in initial assessment and management, with variation between hospitals suggesting scope for learning from high performing centres.

## Supplementary material

10.1136/bmjresp-2025-003207 online supplemental file 1

## Data Availability

The data that support the findings of this study are available from the corresponding author, (CA), upon reasonable request.
