# Supplementary material for: Performance against quality indicators in the initial assessment of patients with respiratory infections in acute medicine services
Source: BMJ Open Respir Res. 2025 Aug 26;12(1):e003207. doi: 10.1136/bmjresp-2025-003207 (PMC12382532; doi:10.1136/bmjresp-2025-003207)
Supplement: online supplemental file 1 [file bmjresp-12-1-s001.docx]

| **Patient level respiratory infection questions**   - Was this presentation due to a respiratory problem? - Was this patient suspected to have a respiratory infection? - If yes, was this:   CAP/Respiratory virus (confirmed result)/Other LRTI/Infective exacerbation of chronic lung disease/HAP   - If CAP, was a CURB65 score documented in the patient notes? - *If yes, what was the CURB65 score?*   0/1/2/3/4/5   - Was oxygen prescribed? - Was the patient given supplemental oxygen (prior to consultant assessment)? - Were antibiotics prescribed?   *If yes, was the first dose IV?*   - *If yes, time from hospital arrival to first dose of antibiotics*   <1hour/1-2hours/2-4hours/4-8hours/>8hours/Unknown   - Did the patient have a chest x-ray? - *If yes, time from hospital arrival to chest x-ray*   <1hour/1-2hours/2-4hours/4-8hours/8-12hours/>12hours/Unknown  *Was a formal report available within 12hrs of the chest x-ray?*   - At 7 days: Was the patient seen by a Respiratory specialist during admission?   **Hospital level respiratory infection questions**   - Does your hospital have a separate respiratory ‘take’/admission service?   Yes – all hours/Yes – selected hours/No   - Do you have local guidelines for Community Acquired Pneumonia?   Yes – specific guideline/As part of wider antibiotic/infection guidelines/No   - Does your unit use a Community Acquired Pneumonia care bundle? - Do you provide written patient information for Community Acquire Pneumonia for patients discharged from AMU? - Do you have point of care testing for Influenza? - Do you have point of care testing for COVID-19? - Do you have point of care testing for any other respiratory viruses? |
| --- |

Supplementary box 1: Questions included in wSAMBA24 specific to respiratory infection. Answer options for all questions were Yes/No/Unknown unless otherwise specified. CAP: community acquired pneumonia; LRTI: lower respiratory tract infection; HAP: hospital acquired pneumonia. IV: intravenous; AMU: acute medical unit.


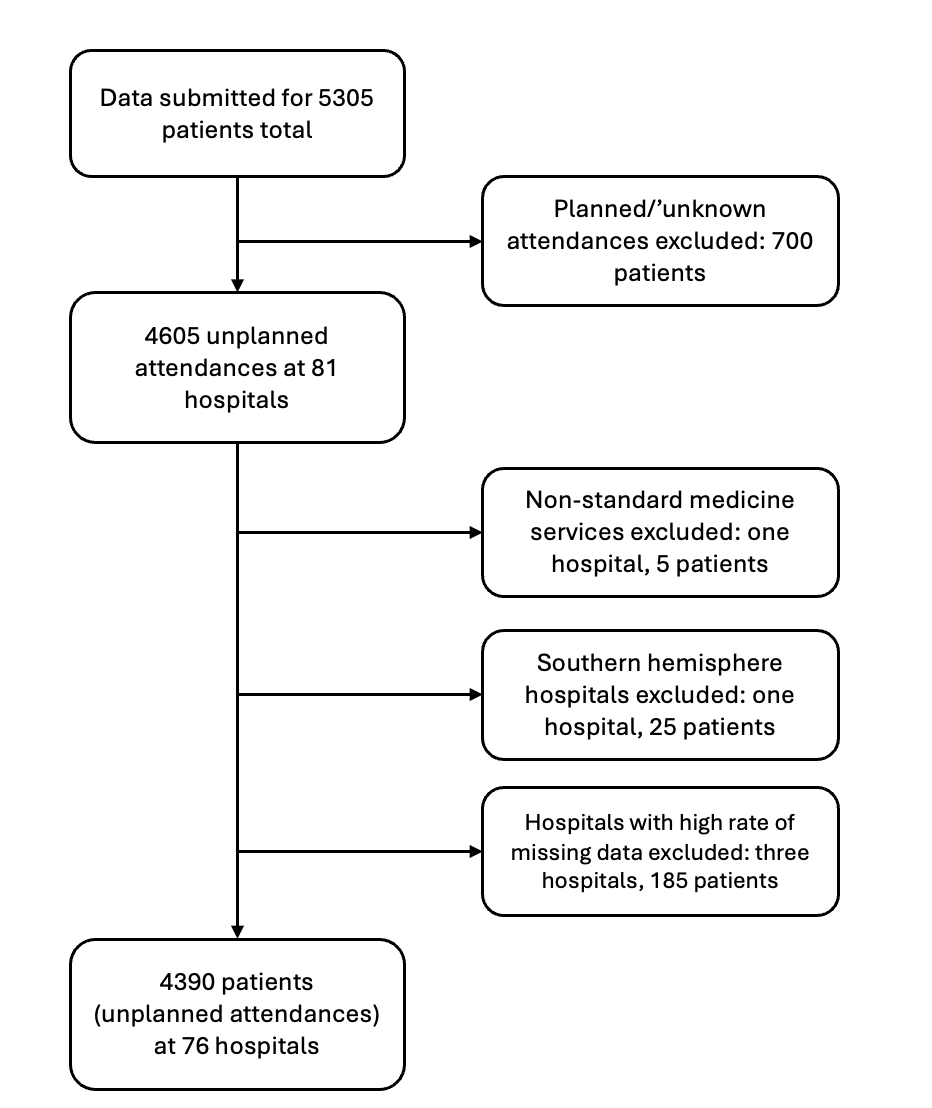


Supplementary Figure 1: Flow diagram of patient inclusion/exclusion.

|  | CAP | | LRTI | | Infective exacerbation | | Respiratory virus | | HAP | | P value |
| --- | --- | --- | --- | --- | --- | --- | --- | --- | --- | --- | --- |
|  | % | N | % | N | % | N | % | N | % | N |  |
| Age  16-19  20-29  30-39  40-49  50-59  60-69  70-79  80-89  90+  *Missing*  Aged ≥70 years | 0.8%  2.7%  5.1%  5.1%  7.0%  15.6%  27.2%  25.3%  11.3%  63.7% | 3/372  10/372  19/372  19/372  26/372  58/372  101/372  94/372  42/372  237/372 | 1.2%  3.7%  6.1%  7.1%  11.0%  16.0%  25.5%  22.4%  7.1%  54.9% | 4/326  12/326  20/326  23/326  36/326  52/326  83/326  73/326  23/326  179/326 | 0.5%  0.9%  0.9%  4.5%  13.5%  23.0%  32.0%  22.5%  2.3%  56.8% | 1/222  2/222  2/222  10/222  30/222  51/222  71/222  50/222  5/222  *1*  126/222 | 0%  6.1%  6.1%  4.6%  15.2%  18.2%  16.7%  18.2%  15.2%  50.0% | 0/66  4/66  4/66  3/66  10/66  12/66  11/66  12/66  10/66  33/66 | 0  3.7%  0  3.7%  3.7%  18.5%  29.6%  25.9%  14.8%  70.4% | 0/27  1/27  0/27  1/27  1/27  5/27  8/27  7/27  4/27  19/27 | 0.015^$^  0.041* |
| Gender  Male (%)  *Missing* | 45.4% | 169/372 | 43.3% | 141/326 | 41.0% | 91/222  *1* | 39.4% | 26/66 | 40.7% | 11/27 | 0.726* |
| Care home residence  *Missing* | 11.1% | 41/371  *1* | 9.9% | 32/325  *1* | 4.1% | 9/223 | 7.6% | 5/66 | 25.9% | 7/27 | 0.007* |
| Hospital discharge within previous 30 days  *Missing* | 20.8% | 77/371  *1* | 17.2% | 56/325  *1* | 27.2% | 60/221  *2* | 21.2% | 14/66 | 88.9% | 24/27^ | <0.001*  (0.05 when HAP excluded) |
| NEWS2 on arrival  0  1  2  3  4  5  6  7+  *Missing*  ≥3 | 8.5%  10.9%  12.4%  13.2%  13.8%  10.6%  7.1%  23.5%  68.2% | 29/340  37/340  42/340  45/340  47/340  36/340  24/340  80/340  *32*  232/340 | 14.9%  14.2%  19.1%  11.9%  10.9%  7.6%  7.3%  14.2%  51.8% | 45/303  43/303  58/303  36/303  33/303  23/303  22/303  43/303  *23*  157/303 | 8.0%  8.5%  9.0%  15.9%  13.9%  10.0%  10.0%  24.9%  74.6% | 16/201  17/201  18/201  32/201  28/201  20/201  20/201  50/201  *22*  150/201 | 11.7%  10.0%  10.0%  15.0%  15.0%  10.0%  8.3%  20.0%  68.3% | 7/60  6/60  6/60  9/60  9/60  6/60  5/60  12/60  *6*  41/60 | 4.6%  9.1%  9.1%  0  9.1%  13.6%  4.6%  50.0%  77.3% | 1/22  2/22  2/22  0/22  2/22  3/22  1/22  11/22  *5*  17/22 | <0.001^$^  <0.001* |
| Daytime arrival  (08:00-19:59) | 77.7% | 289/372 | 76.4% | 249/326 | 72.7% | 162/223 | 57.6% | 38/66 | 74.1% | 20/27 | 0.011* |
| Source of referral  ED  GP  Paramedic  Other | 67.7%  15.1%  11.8%  5.4% | 252/372  56/372  44/372  20/372 | 62.9%  21.8%  9.5%  5.8% | 205/326  71/326  31/326  19/326 | 69.1%  16.1%  9.9%  4.9% | 154/223  36/223  22/223  11/223 | 65.2%  13.6%  15.2%  6.1% | 43/66  9/66  10/66  4/66 | 63.0%  3.7%  22.2%  11.1% | 17/27  1/27  6/27  3/27 | 0.146* |
| Location of first clinical assessment  ED  AMU  SDEC  Other  *Missing* | 84.3%  4.6%  10.8%  0.3% | 213/370  17/370  40/370  1/370  *2* | 71.5%  7.4%  21.1%  0 | 231/323  24/323  68/323  0  *3* | 88.2%  5.0%  6.8% | 194/220  11/220  15/220  *3* | 83.3%  6.1%  10.6% | 55/66  4/66  7/66 | 88.9%  3.7%  7.4% | 24/27  1/27  2/27 | 0.001* |

Supplementary table 1: Comparison of patient characteristics and demographics by respiratory infection type. P value for Chi square test, except where indicated ^$^ for Kruskal-Wallis test. CAP: community acquired pneumonia; LRTI: lower respiratory tract infection; HAP: hospital acquired pneumonia; NEWS2: National Early Warning Score 2; ED: emergency department; GP: general practice; AMU: acute medical unit; SDEC: same day emergency care. ^3/27 patients not recorded as recent hospital discharge; this may reflect admission in a different hospital or in another institution leading to clinical diagnosis of hospital acquired pneumonia.

| **CQI2** | **Odds ratio** | **P value** | **95% Confidence interval** |
| --- | --- | --- | --- |
| Suspected respiratory infection | 0.86 | 0.141 | 0.695-1.053 |
| Arrival time  00:00-03:59  04:00-07:59  08:00-11:59  12:00-15:59  16:00-19:59  20:00-23:59 | Ref  1.55  3.52  2.63  1.51  1.19 | 0.037  <0.001  <0.001  0.004  0.276 | 1.028-1.324  2.594-4.790  1.974-3.492  1.142-1.992  0.869-1.636 |
| NEWS2 score* | 1.16 | <0.001 | 1.111-1.206 |
| Location of initial assessment  ED  AMU  SDEC  Other | Ref  1.25  1.58  0.86 | 0.211  <0.001  0.711 | 0.881-1.772  1.270-1.956  0.384-1.921 |
| **CQI3** | **Odds ratio** | **P value** | **95% confidence interval** |
| Suspected respiratory infection | 1.06 | 0.553 | 0.866-1.308 |
| Arrival time  00:00-03:59  04:00-07:59  08:00-11:59  12:00-15:59  16:00-19:59  20:00-23:59 | Ref  2.01  0.19  0.15  0.03  0.74 | 0.010  <0.001  <0.001  <0.001  0.088 | 1.182-3.425  0.136-0.255  0.114-0.210  0.024-0.048  0.519-1.046 |
| NEWS2 score* | 1.07 | <0.001 | 1.031-1.109 |
| Location of initial assessment  ED  AMU  SDEC  Other | Ref  6.09  19.32  1.80 | <0.001  <0.001  0.233 | 4.295-8.646  14.78-25.25  0.684-4.755 |

Supplementary Table 2: Logistic regression models for likelihood of achieving Clinical Quality Indicator (CQI) target times. CQI2: assessment by competent clinical decision maker within 4 hours of hospital arrival; CQI3: assessment by consultant physician within target time (6 hours for arrivals between 08:00-19:59; 14 hours for arrivals between 20:00-07:59). NEWS2: National Early Warning Score 2; ED: emergency department; AMU: acute medical unit; SDEC: same day emergency care. *NEWS2 odds ratio per point increase in score.

| **Chest x-ray in 4 hours** | **Odds ratio** | **P value** | **95% confidence interval** |
| --- | --- | --- | --- |
| Arrival time  00:00-03:59  04:00-07:59  08:00-11:59  12:00-15:59  16:00-19:59  20:00-23:59 | Ref  1.77  2.02  2.17  1.49  1.36 | 0.204  0.016  0.005  0.153  0.345 | 0.732-4.300  1.141-3.564  1.259-3.749  0.861-2.593  0.719-2.568 |
| NEWS2 score* | 1.172 | <0.005 | 1.103-1.245 |
| Location of initial assessment  ED  AMU  SDEC | Ref  0.37  1.96 | 0.001  0.018 | 0.204-0.686  1.123-3.423 |
| **Antibiotics within 4 hours** | **Odds ratio** | **P value** | **95% confidence interval** |
| Arrival time  00:00-03:59  04:00-07:59  08:00-11:59  12:00-15:59  16:00-19:59  20:00-23:59 | Ref  3.309  2.28  1.73  1.32  1.86 | 0.037  0.013  0.081  0.377  0.102 | 1.074-10.197  1.191-4.381  0.934-3.206  0.710-2.470  0.884-3.920 |
| NEWS2 score* | 1.31 | <0.001 | 1.225-1.411 |
| Location of initial assessment  ED  AMU  SDEC | Ref  0.38  1.05 | 0.007  0.887 | 0.189-0.767  0.546-2.015 |

Supplementary table 3: Logistic regression model for achievement of four-hour targets for chest x-ray and antibiotic delivery. NEWS2: National Early Warning Score; ED: Emergency department; AMU: acute medical unit; SDEC: same day emergency care. *NEWS2 odds ratio per point increase in score.

|  | Overall | | | | | Hospital-level performance | | | |
| --- | --- | --- | --- | --- | --- | --- | --- | --- | --- |
|  | CAP | | All non-CAP diagnoses | |  | CAP | | All non-CAP diagnoses | |
|  | % | N | % | N | P value | Median | IQR (range) | Median | IQR (range) |
| CURB65 documented* | 37.9% | 139/367 | n/a | n/a |  | 37.5% | 0-50.0%  (0-100%) | n/a | n/a |
| Chest x-ray within 4 hours^ | 70.0% | 248/354 | 71.6% | 403/563 | 0.62 | 75.0% | 50.0-100%  (0-100%) | 75.0% | 61.9-94.6%  (25.0-100%) |
| Chest x-ray report within 12 hours^ | 26.3% | 94/357 | 27.5% | 155/564 | 0.846 | 20.0% | 0-40.0%  (0-100%) | 16.7% | 0-50.0%  (0-100%) |
| Antibiotics within 4 hours | 67.3% | 214/318 | 67.0% | 282/421 | 0.929 | 75.0% | 50.0-100%  (0-100%) | 75.0% | 50-100%  (0-100%) |
| Prescription for administered oxygen | 71.2% | 148/208 | 62.6% | 169/270 | 0.05 | 100% | 50.0-100%  (0-100%) | 92.9% | 45.8-100%  (0-100%) |

Supplementary Table 4: Performance against standards for management of Community Acquired Pneumonia (CAP), comparing patients with CAP to all other respiratory infection diagnoses. Performance shown for all included patients, with p value for comparison between patients with CAP and those with diagnoses other than CAP, and for performance by hospital. *CURB65 score only documented for patients with CAP. ^Target applied to patients who had chest x-ray performed. IQR: interquartile range

| Same day discharge | Odds ratio | P value | 95% Confidence interval |
| --- | --- | --- | --- |
| Respiratory infection  None  CAP  Other LRTI  Infective exacerbation  Respiratory virus  HAP | Ref  0.35  1.34  0.33  0.66  N/A | <0.001  0.078  <0.001  0.369 | 0.222-0.540  0.968-1.851  0.174-0.612  0.267-1.633 |
| Arrival time  00:00-03:59  04:00-07:59  08:00-11:59  12:00-15:59  16:00-19:59  20:00-23:59 | Ref  1.07  1.46  0.97  0.48  0.11 | 0.767  0.020  0.874  <0.001  <0.001 | 0.674-1.708  1.060-2.005  0.709-1.340  0.345-0.677  0.065-0.193 |
| NEWS2 score | 0.66 | <0.001 | 0.623-0.698 |
| Age  16-19  20-29  30-39  40-49  50-59  60-69  70-79  80-89  90+ | Ref  1.82  1.72  1.63  1.13  0.90  0.60  0.33  0.25 | 0.080  0.108  0.150  0.720  0.745  0.114  0.001  <0.001 | 0.930-3.568  0.888-3.324  0.839-3.151  0.591-2.143  0.474-1.706  0.317-1.131  0.173-0.630  0.119-0.543 |
| Referral source  Emergency Department  GP  Paramedic  Other | Ref  3.06  0.57  1.82 | <0.001  0.003  <0.001 | 2.503-3.734  0.393-0.823  1.379-2.396 |
| Recent discharge from hospital (30 days) | 0.61 | <0.001 | 0.486-0.755 |

Supplementary Table 5: Logistic regression model for likelihood of same day discharge. Pseudo R2=0.269. CAP: community acquired pneumonia; LRTI: Lower respiratory tract infection; HAP: hospital acquired pneumonia; NEWS2: National Early Warning Score 2; GP: general practice.
